# The First Lethal Infection by *Oligella ureolytica*: A Case Report and Review of the Literature

**DOI:** 10.3390/antibiotics12091470

**Published:** 2023-09-21

**Authors:** Pierre Serandour, Chloé Plouzeau, Anthony Michaud, Lauranne Broutin, Julie Cremniter, Christophe Burucoa, Maxime Pichon

**Affiliations:** 1 Université de Poitiers, Faculté de Médecine et Pharmacie, 86000 Poitiers, France; 2 CHU de Poitiers, Département des Agents Infectieux, 86021 Poitiers, France; 3 Université de Poitiers, INSERM U1070, Pharmacologie des Agents Antimicrobiens et Antibiorésistance, 86022 Poitiers, France

**Keywords:** *Oligella ureolytica*, *Alcaligenaceae*, Gram negative bacillus, 16S rDNA sequencing, Sanger sequencing

## Abstract

*Oligella ureolytica* is a Gram-negative bacillus, a member of the *Alcaligenaceae* family, that had never previously been reported as lethal. Herein, a case of fatal infection caused by *Oligella ureolytica* in an elderly woman with suspected bladder cancer is reported. The species identification was confirmed through Sanger sequencing of the bacterial 16S rRNA sequence and compared to published sequences for phylogenetic analysis. Initial antibiotic therapy with ceftriaxone and oxacillin was initiated but had to be switched due to resistance. Cefepime in combination with metronidazole was administered, unfortunately failing to prevent the patient’s death. Further studies are needed to explore additional factors influencing clinical outcomes in *Oligella ureolytica* infections.

## 1. Introduction

Discovered and described in scientific literature in 1990, *Oligella ureolytica* is a Gram-negative bacteria that belongs to the *Oligella* genus, which is a member of the *Alcaligenaceae* family [1]. The *Alcaligenaceae* family consists of Gram-negative bacteria that are widely distributed in natural environments such as soil, water, and plants [1]. As these bacteria are known for their metabolic versatility, particularly their ability to metabolize a wide range of organic compounds, they are found in both the environment and in clinical settings, where they can act as opportunistic pathogens. The *Oligella* genus, so named because of the small size of the bacilli on Gram stain, has been isolated from a variety of clinical samples and associated with infections such as those of the urinary tract, encompassing several species, including *Oligella urethralis* and *Oligella ureolytica* [2]. These bacteria have attracted attention due to their clinical importance and their association with various infections in humans. Understanding the characteristics and distinctions between these species is essential for accurate diagnosis and effective management of *Oligella*-related infections. *Oligella urethralis*, formerly classified as *Moraxella urethralis* or Centers for Disease Control and Prevention (CDC) group M-4, is a species of the genus *Oligella* that causes urinary tract infections, respiratory tract infections, and septicemia [2]. On the other hand, *Oligella ureolytica*, another member of the genus *Oligella* previously known as group CDC IVe, is a Gram-negative, aerobic, motile bacterium with peritrichous flagella of the genus *Oligella*, most isolated from human urine, especially in contexts of ongoing neoplasia and urinary obstruction when patients require long-term urinary catheters or other urinary drainage catheters [3]. *O. ureolytica* is characterized by its ability to produce urease, an enzyme that hydrolyses urea into ammonia and carbon dioxide, thereby contributing to its survival and colonization and leading to its implication in a variety of infections, including urinary tract infections, wound infections, and respiratory tract infections [2].

This clinical case presents an 87-year-old woman who was admitted to the hospital with a deteriorating general condition, weight loss (associated with severe malnutrition), and a septic syndrome (of urinary origin and suspected of being associated with bladder cancer, but this was not confirmed due to the absence of further investigations). Despite appropriate therapies and prolonged antibiotic treatment, the septic syndrome could not be controlled, leading to the patient’s death three weeks after hospitalization.

This article aimed to provide an overview of *Oligella ureolytica*, exploring its characteristics, clinical significance, and relevant research findings. By examining the clinical case and reviewing the existing literature, it should enhance understanding of *Oligella ureolytica* infections, helping to improve diagnosis, management, and prevention strategies for this clinically emerging pathogen.

## 2. Case Report

An 87-year-old woman with a history of heart failure and suspected bladder failure (no further investigations) was admitted on 15 May 2020 (D0) to the Montmorillon Emergency Department (Poitiers University Hospital, France) with progressive deterioration in her general condition, severe malnutrition, and a septic syndrome of urinary origin. The clinical presentation included a heart rate of 61 bpm, blood pressure of 121/63 mmHg, respiratory rate of 21/min, and oxygen saturation of 96% on room air, without fever (36.9 °C). On her arrival at the hospital, her biological work-up showed a significant rise in the leucocyte count (34 × 10^9^/L) and anemia (hemoglobin 8.7 g/dL), associated with a biological inflammatory syndrome (elevated inflammatory markers, including CRP 49 mg/L). She also had acute renal failure (creatinine 158 μmol/L) with hyponatremia (130 mmol/L), hyperkalemia (5.2 mmol/L), and hypercalcemia (3 mmol/L). Urine dipstick was positive for leukocytes and nitrites. Uroculture was performed according to the recommendations of the French Society of Microbiology using chromogenic media (UTI agar plates, Thermo Scientific, Waltham, MA, USA) incubated for 24 h at 35 °C in an aerobic environment.

Given the clinical picture, ceftriaxone iv. associated with oxacillin iv. was started immediately. Clinical examination revealed no fever, but rather an infection of the left lung, because of which the antibiotic therapy was changed to amoxicillin-clavulanate (empirically in the absence of bacteriological analysis). As for hypercalcemia, the low PTH level led to the conclusion that it was due to a secondary cause, probably neoplasia (consistent with the hypothesis of urinary neoplasia). Given the association of anemia, hypercalcemia, and renal failure, plasma protein electrophoresis was performed, ruling out myeloma. A CT scan showed very significant thickening of the bladder walls, which measured between 24 mm (anterior wall) and 38 mm (posterolateral right wall) in maximum thickness, associated with significant right ureterohydronephrosis, as well as a right inguinal hernia containing possible adenopathy. This examination revealed an osteolytic lesion at the pubic symphysis, right iliopubic branch, confirming the neoplastic origin initially suspected, as the sole cause of possible immunosuppression.

**Figure 1:**
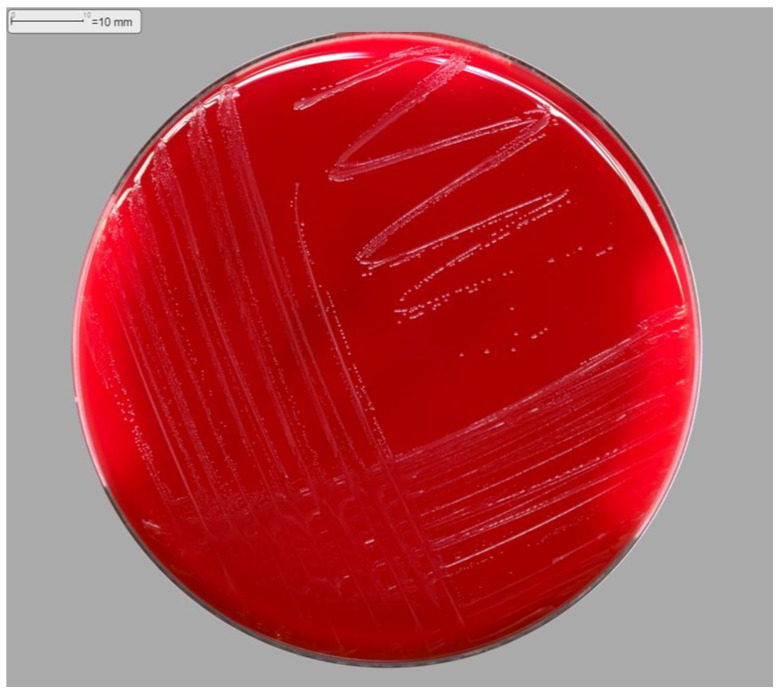
Colonies of *Oligella Ureolytica* isolated on fresh blood agar.

**Figure 2:**
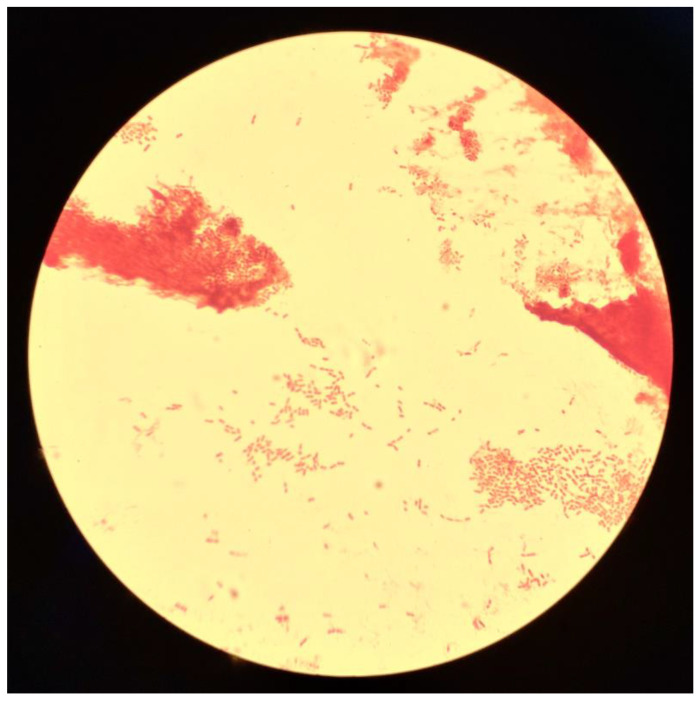
Direct examination after gram staining of *Oligella urolytica* (×1000 magnification).

On day 1, the bacteriological culture of the blood culture revealed *Oligella* sp., a genus that is frequently sensitive to beta-lactams, leading to the continuation of probabilistic antibiotic therapy pending antibiotic susceptibility testing (Figure 1 and Figure 2). In this context, no specific exposure to natural environments (soil, contaminated water, and plants) was collected in the patient’s history.

On the third day (D3), the antibiogram was determined using the disk diffusion method and eTests (bioMérieux, Marcy-l’étoile, France), in accordance with CA-SFM/EUCAST recommendations at the time of diagnosis (Table 1). The AST profile revealed multi-susceptibility to several antibiotics tested, cefepime, imipenem, meropenem, tobramycin, amikacin, gentamicin, cotrimoxazole and rifampicin, and with six resistance profiles (ticarcillin, ticarcillin clavulanic acid, piperacillin + tazobactam (MIC 48 mg/L), ceftriaxone (lack of initial efficacy), aztreonam, and ceftazidime (MIC > 256 mg/L)) and two intermediate profiles (amoxicillin and amoxicillin-clavulanate) that could explain the slow improvement with amoxicillin-clavulanate.

On the fourth day (D4), considering the antibiogram of the bacteria found in the blood cultures, the patient was started on cefepime iv. and metronidazole iv. to obtain good anaerobic coverage given the associated inhalation pneumonitis. Cefepime was initiated at a dose of 2 g in the evening, modified at D7 to a dose of 1 g twice daily given the patient’s renal clearance (dosages of antibiotics were not carried out in the present case). Follow-up blood cultures were taken on D7 but remained negative after culture.

Antibiotic therapy was stopped on D11, but the thick urine persisted, so a 24-h break was taken before changing the catheter and checking the uroculture (as described previously) before reintroducing antibiotic therapy. On D12, biological tests revealed a further increase in hyperleukocytosis and thrombocytosis. Given the major dilatation of the urinary tract and the persistence of infectious signs, there was a theoretical indication for a urinary diversion, but given the patient’s general condition and end-of-life situation, she was not eligible for this type of treatment, and comfort care was preferred. Despite targeted antibiotic therapy, the patient died on D17.

The final retrospective identification of the organism involved as *Oligella ureolytica* was obtained by Sanger sequencing of the V1 to V3 regions of the 16S rDNA.

## 3. Discussion

To the best of our knowledge, the present article is the first description of a fatal case of *Oligella ureolytica* infection, and the first case reported in France (Table 2). The emergence of *O. ureolytica* as a clinically significant pathogen is of interest. Firstly, accurate identification of *O. ureolytica* is crucial for appropriate diagnosis and management. Historically, misidentification of this bacterium with other related species, such as *Alcaligenes faecalis*, has been common [4]. However, advances in molecular techniques, such as polymerase chain reaction (PCR) and matrix-assisted laser desorption/ionization time-of-flight mass spectrometry (MALDI-TOF MS), have improved the accuracy of identification. *O. ureolytica* has been implicated in a variety of infections, including urinary tract infections, wound infections, and respiratory tract infections.

An important role in the pathogenesis of *O. ureolytica* is played by the production of urease. Urease is an enzyme that catalyzes the hydrolysis of urea, leading to the production of ammonia and carbon dioxide. These compounds affect the surrounding environment via their alkalization [3]. The pH alkaline favors bacterial survival and colonization, contributing to the persistence and severity of infections. The formation of biofilms by *O. ureolytica* further complicates infection management [4]. Biofilms are structured communities of bacteria enclosed in a protective extracellular matrix. The presence of biofilms confers increased resistance to antibiotics and host immune responses, which complicates the eradication of *O. ureolytica* infections. Future research should focus on understanding the mechanisms underlying biofilm formation and developing strategies to disrupt or prevent biofilm formation, the objective being to improve treatment outcomes in these contexts.

The clinical significance of *O. ureolytica* infections is not well-established, partly due to a lack of awareness and limited data on their prevalence. Further epidemiological studies are needed to determine the true incidence and prevalence of *O. ureolytica* infections and to identify associated potential risk factors and clinical outcomes. In addition, studies on the virulence factors and pathogenic mechanisms of *O. ureolytica* could provide valuable information on its pathogenesis and contribute to the development of targeted therapeutic interventions.

In terms of treatment, *O. ureolytica* infections must be treated on a case-by-case basis, considering the site and severity of the infection. While empirical treatment with broad-spectrum antibiotics is often initiated, susceptibility testing should guide the selection of appropriate antimicrobial agents. It is important to note that *O. ureolytica* can exhibit multi-drug resistance, particularly in the context of biofilm formation. Therefore, a combination of antimicrobial agents or other therapeutic approaches may be required to effectively eradicate these infections.

## 4. Conclusions

This case of *Oligella ureolytica* in an elderly woman with possible bladder cancer underscores the importance of sequencing new strains of *Oligella* spp to reference pathogenic strains of a genus such as *Oligella*, which may be predominantly described in clinical infections. *O ureolytica* is an emerging pathogen of the *Alcaligenaceae* family, associated with a range of infections in humans. Accurate identification, understanding of its pathogenic mechanisms, and appropriate management are essential to improved patient outcomes. Further research is needed to deepen our knowledge of this bacterium, including its epidemiology, virulence factors, and optimal treatment strategies, which will contribute to the prevention, diagnosis, and management of *O. ureolytica* infections in the clinical setting.

## Figures and Tables

**Table 1:** Antibiotic susceptibility profile (AST) of *O. ureolytica* using the disk diffusion method and e-Test. S = susceptible, I = intermediate, R = resistant; MIC: minimum inhibitory concentration.

Antibiotic Tested	Categorization	MIC (mg/L)
Amoxicillin	I	
Amoxicillin + Clavulanic acid	I	
Ticarcillin	R	
Ticarcillin + Clavulanic acid	R	
Piperacillin Tazobactam (e-test)	R	48
Ceftriaxone	R	
Ceftazidime (e-test)	R	>256
Cefepime	S	
Imipenem	S	
Meropenem	S	
Aztreonam	R	
Tobramycin	S	
Amikacin	S	
Gentamicin	S	
Cotrimoxazole	S	
Rifampicin	S	

**Table 2:** Published cases of *O. Ureolytica* infections.

Year	Age/Sex	Culture Source	Underlying Conditions	Outcome	AST Profile	Reference
2023	87 W	Bloodstream infection	Vesical neoplasia	Died on D17	Table 1	Present Case
2023	24/M	Deep brain stimulator (DBS)	Tourette syndrome	Cured by antimicrobial therapy, (vancomycin and cefepime for 6 weeks), reimplantation of the DBS	Susceptible to amikacin, ampicillin, aztreonam, cefepime, cefoxitin, ceftazidime, gentamicin, meropenem, P-T, tetracycline, tobramycin, T-S Intermediary resistant to erythromycin Resistant to ciprofloxacin, clindamycin, levofloxacin, linezolid, rifampin	[2]
2020	-	Tattoo and permanent makeup inks	-	-	-	[5]
2019	-	Automated MRI contrast injectors	-	-	-	[6]
2016	66/M	Bloodstream infection	Heart surgical intervention of aortic valve substitution with bio-prosthesis	Cured by antimicrobial therapy (vancomycin, gentamicin, and rifampin switched for piperacillin/tazobactam for 14 days)	Susceptible to amoxicillin/clavulanic acid, piperacillin/tazobactam, levofloxacin and penems Intermediately resistant to erythromycin Resistant to ampicillin and 3rd generation cephalosporins	[7]
2015	66/W	Bloodstream infection	Femur fracture, right buttock stage 3 decubitus ulcer	Cured by antimicrobial therapy (vancomycin, aztreonam, and metronidazole for 10 days)	Susceptible to amikacin, ampicillin/sulbactam, ceftazidime, ceftriaxone, gentamicin, imipenem, levofloxacin, nitrofurantoin, trimethoprim/sulfamethoxazole, chloramphenicol. No resistance	[8]
2014	Newborn/ W	Bloodstream infection	Weakness in reflexes, Icterus	Cured by antimicrobial therapy (netilmicin for 10 days)	Susceptible to amoxicillin-clavulanic acid, gentamicin, cefuroxime, ceftriaxone, ceftazidime, ciprofloxacin, carbapenems Intermediate resistance to ampicillin and Resistant to cotrimoxazole	[3]
2014	30/M	Bloodstream infection	Right lung adenocarcinoma, Multiple abdominal lymph node metastases, Syringomyelia	Cured by antimicrobial therapy (amoxicillin and clavulanic acid, meropenem. The patient became afebrile after 3 days)	Susceptible to imipenem and meropenem Resistant to ampicillin, piperacillin, piperacillin/tazobactam, tobramycin, amikacin, ciprofloxacin, trimethoprim-sulfamethoxazole (TMP-SMX), ceftazidime, ceftriaxone	[9]
1996	49/W	Cervical lymph node	Chronic lymphocytic leukemia	Cured by antimicrobial therapy (ciprofloxacin for 7 days followed by TMP-SMX for 2 weeks, then cephalexin (an antibiotic to which the organism was resistant in vitro) and chemotherapy)	Susceptible to aminoglycosides and cephalosporins Resistant to usual serum concentrations of ampicillin chloramphenicol, erythromycin, penicillin G, tetracycline and TMP-SMX	[10]
1993	40/M	Bloodstream infection	AIDS, Chronic diarrhea, Kaposi’s sarcoma, Thrush	Cured by antimicrobial therapy (IV tobramycin, oral ciprofloxacin, and fluconazole) died secondary to fungemia	Susceptible to gentamicin, tobramycin, imipenem/cilastatin. TMP SMZ. and ciprofloxacin Resistant to aztreonam, mezlocillin, ticarcillin/clavulanic acid, piperacillin. and ceftazidime.	[11]
1990	5	Pneumonia	Chronic granulomatous disease	Cured by antimicrobial therapy (after a 2-week trimethoprim-sulfamethoxazole intravenous antibiotic course, he was discharged from the hospital on oral trimethoprim- sulfamethoxazole. All symptoms of pulmonary disease had resolved at the time of a 2-month follow-up visit)	Susceptible to trimethoprim-sulfamethoxazole, gentamicin, tobramycin, amikacin, imipenem, and ciprofloxacin. Resistant to chloramphenicol, ampicillin, mezlocillin, aztreonam, and all cephalosporins tested, including ceftazidime.	[1]

## Data Availability

Not applicable.

## References

[1] Trotter J.A., Kuhls T.L., Pickett D.A., Reyes de la Rocha S., Welch D.F. (1990). Pneumonia Caused by a Newly Recognized Pseudomonad in a Child with Chronic Granulomatous Disease. J. Clin. Microbiol..

[2] Edwards M.K., Kollu V., Kalyatanda G.S. (2023). Deep Brain Stimulator Infection by Oligella: A Case Report and Review of the Literature. Cureus.

[3] Demir T., Celenk N. (2014). Bloodstream Infection with Oligella Ureolytica in a Newborn Infant: A Case Report and Review of the Literature. J. Infect. Dev. Ctries..

[4] Rossau R., Kersters K., Falsen E., Jantzen E., Segers P., Union A., Nehls L., De Ley J. (1987). *Oligella*, a New Genus Including *Oligella urethralis* Comb. Nov. (Formerly *Moraxella urethralis*) and *Oligella ureolytica* Sp. Nov. (Formerly CDC Group IVe): Relationship to *Taylorella equigenitalis* and Related Taxa. Int. J. Syst. Bacteriol..

[5] Nho S.W., Kim M., Kweon O., Kim S.-J., Moon M.S., Periz G., Huang M.-C.J., Dewan K., Sadrieh N.K., Cerniglia C.E. (2020). Microbial Contamination of Tattoo and Permanent Makeup Inks Marketed in the US: A Follow-up Study. Lett. Appl. Microbiol..

[6] Goebel J., Steinmann J., Heintschel von Heinegg E., Hestermann T., Nassenstein K. (2019). Bacterial Contamination of Automated MRI Contrast Injectors in Clinical Routine. GMS Hyg. Infect. Control.

[7] Pagotto A., Merluzzi S., Pillinini P., Valeri M. (2016). Bloodstream Infection with *Oligella ureolytica*: A Case Report and Review of the Literature. Infez. Med..

[8] Simmons T., Fennelly E., Loughran D. (2015). Oligella Ureolytica Bacteremia in Elderly Woman, United States. Emerg. Infect. Dis..

[9] Baruah F.K., Jain M., Lodha M., Grover R.K. (2014). Blood Stream Infection by an Emerging Pathogen Oligella Ureolytica in a Cancer Patient: Case Report and Review of Literature. Indian J. Pathol. Microbiol..

[10] Baqi M., Mazzulli T. (1996). Oligella Infections: Case Report and Review of the Literature. Can. J. Infect. Dis. Med. Microbiol..

[11] Manian F.A. (1993). Bloodstream Infection with Oligella Ureolytica, Candida Krusei, and Bacteroides Species in a Patient with AIDS. Clin. Infect. Dis..

